# Quantitative Assessment of Ultraviolet-Induced Erythema and Tanning Responses in the Han Chinese Population

**DOI:** 10.1007/s43657-023-00105-1

**Published:** 2023-06-05

**Authors:** Yanyun Ma, Yimei Tan, Yue Hu, Weilin Pu, Jinhua Xu, Li Jin, Jiucun Wang

**Affiliations:** 1 State Key Laboratory of Genetic Engineering, School of Life Sciences, and Human Phenome Institute, Fudan University, Shanghai, 201203 China; 2 Six-Sector Industrial Research Institute, Fudan University, Shanghai, 200433 China; 3 Shanghai Skin Disease Hospital, Shanghai, 200443 China; 4 Department of Dermatology, Huashan Hospital, Fudan University, Shanghai, 200040 China; 5 MOE Key Laboratory of Contemporary Anthropology, School of Life Science, Collaborative Innovation Center for Genetics and Development, Fudan University, Shanghai, 200438 China; 6 Research Unit of Dissecting the Population Genetics and Developing New Technologies for Treatment and Prevention of Skin Phenotypes and Dermatological Diseases, Chinese Academy of Medical Sciences (2019RU058), Shanghai, 200438 China

**Keywords:** Ultraviolet radiation, Erythema response, Tanning ability, Han Chinese population

## Abstract

Ultraviolet radiation (UVR) can induce erythema and tanning responses with strong diversity within and between populations, but there were no precise method for evaluating the variation in these responses. In this study, we assessed the time course of ultraviolet (UV)-induced responses based on the erythema index (EI) and melanin index (MI) over 14 consecutive days in a pilot cohort study (N = 31). From safety evaluations, we found that no skin blisters occurred at a UV dosage of 45 mJ/cm^2^, but there were significant skin reactions. Regardless of UV dosage, the measurements and variances of EI peaked on day 1 after UV irradiation, and those of MI peaked on day 7. Dose–response curves, including erythema dose–response (EDR) and melanin dose–response (MDR), could measure UV-induced phenotypes sensitively but more laboriously. As an alternative, we directly represented the UV-induced erythema and tanning responses using the erythema increment (ΔE) and melanin increment (ΔM). We found that ΔE and ΔM at 45 mJ/cm^2^ significantly correlated with erythema dose–response (EDR) (*R*^2^ > 0.9) and melanin dose–response (MDR) (*R*^2^ > 0.9), respectively. Therefore, ΔE and ΔM on day 1 and day 7 after UV irradiation at a dosage of 45 mJ/cm^2^ might be ideal alternative measures for assessing individual erythema and tanning responses. Then, a second cohort (N = 664) was recruited to validate the UV-induced phenotypes, and, as expected, the results of the two cohorts were in agreement. Therefore, we developed a simplified and precise method to quantify the UV-induced erythema response and tanning ability for the Han Chinese population.

**Supplementary Information:**

The online version contains supplementary material available at 10.1007/s43657-023-00105-1.

## Introduction

Ultraviolet (UV) light contributes significantly to the increased prevalence of skin cancers and accelerated skin aging (Fry and Ley 1989; Kammeyer and Luiten 2015; Leiter and Garbe 2020; Narayanan et al. 2010; Rittie and Fisher 2015; Rundel and Nachtwey 1978). After exposure to ultraviolet radiation (UVR), skin can both react immediately (erythema response) and after a delay (tanning response) (Jablonski and Chaplin 2010; Park et al. 1998). The erythema response is a cutaneous inflammatory reaction that represents the extent of skin damage. However, skin edema and blisters caused by UVR are serious adverse reactions that can easily lead to skin diseases (Guan et al. 2017; Narayanan et al. 2010). The tanning response involves melanin synthesis to prevent further DNA damage caused by UVR, and has allowed humans to evolve to be more adaptable to the changing UV environment, resulting in a stronger competitive advantage (Jablonski and Chaplin 2010; Narayanan et al. 2010). Unfortunately, there are currently no reliable methods to precisely quantify the two UV-induced phenotypes of erythema and tanning.

Currently, the Fitzpatrick photo-type scale and the minimum erythema dosage (MED) are two widely used parameters representing the extent of UV-induced skin responses. However, these two subjective methods cannot effectively distinguish UV-induced tanning phenotypes from constitutive skin color (Choe et al. 2006; Leenutaphong 1995; Noda et al. 1993; Park et al. 2002; Sanclemente et al. 2008; Wee et al. 1997). The erythema dose–response (EDR) and melanin dose–response (MDR) measurements are based on reflectance spectroscopy, which are regarded as objective measures for the quantification of erythema and tanning responses (Wagner et al. 2002a; Westerhof et al. 1990). However, measuring EDR and MDR is laborious in large-scale population studies (Wagner et al. 2002a, b), because a series of UV doses are required for each individual to calculate the slopes of the dose–response curves of EDR and MDR.

Both erythema and tanning responses are signs of UV-induced skin damage. However, excessive UV is highly likely to induce serious adverse skin reactions, such as blisters and edema, which will reduce the accuracy of measuring UV-induced phenotypes. In addition, the EDR and MDR measurements are usually taken on D1 (day 1) and D7 (day 7) after UV irradiation based on clinical experience. Therefore, a simplified, objective, and accurate measurement technique is needed to construct a standard protocol to measure UV-induced erythema and tanning responses. In this study, we explored the safe UV exposure dose, confirmed the optimal test time points and parameters for assessing UV-induced phenotypes through consecutive measurements, and developed a set of procedures for UV-induced phenotype quantification. Then, we applied this method in an independent cohort for further verification.

## Materials and Methods

### Study Subjects

Participants were recruited between 2013 and 2019. To avoid the interference of sunburn in summer, we implemented all studies between March and May. A total of 695 Han Chinese participants, including 190 males and 505 females, were enrolled. From this group, 31 participants were included in cohort 1 and participated in a 14-consecutive-day study. Subsequently, the remaining 664 participants were included in cohort 2 to complete the validation study by measuring one and seven days after UV exposure. The demographic information of the samples in cohort 1 are shown in Table S1. Written informed consent was obtained from each study participant. The study was approved by the institutional ethics committee of Fudan University (Shanghai, China).

### Clinical Methods

We employed a solar simulator (Multiport^®^ Simulator, Solar Light Co, Philadelphia, PA, USA) to irradiate the back skin of each participant to study UV-induced skin phenotypes. The simulator generates light in the band 290–400 nm, excluding all radiation lower than 290 nm and limiting the radiation above 400 nm to less than 2% of the total output. In cohort 1, the simulator was utilized to administer UV dosages of 36, 40, 45, 50, 55, and 60.0 mJ/cm^2^. In cohort 2, the UV dosages were set as 20, 25, 30, 36, 40, and 45 mJ/cm^2^. To study the erythema and tanning responses, we recorded erythema index (EI) and melanin index (MI) each day from D1 to D14 after UV irradiation. In cohort 2, we completed skin measurements of erythema and tanning responses one and seven days after UV irradiation, respectively.

The skin color on the back of each participant was measured with a colormeter (DSM II colormeter, Cortex Technology, Hadsund, Denmark). DSM II is a small handheld device with two white light-emitting diode (LED) lights to provide an easy selection of different color systems, and calibration can be completed in seconds with the supplied calibrator. A special lens arrangement focused on the target area of 7 mm in diameter significantly reduces the influence of ambient light. In this study, we selected EI and MI to estimate UV-induced erythema and tanning responses, respectively. An un-irradiation site on the back of each participant was measured as a baseline.

Skin blisters and edema were clinically assessed 24 h after UV irradiation by two experienced dermatologists.

### Statistical Methods

Both EI and MI of each skin site were measured three times repeatedly and averaged as the measurements for the site. The EI and MI were used to estimate the redness and melanin concentration of skin, respectively. The individuals with higher EI and MI values have darker redness and tanning pigmentation, respectively.

The EDR was calculated as the slope of EI versus UV dosage on D1, as shown below:

$$\mathrm{EDR }= \frac{\sum_{j=0}^{n}({UV}_{j} - \overline{UV})({EI}_{j} - \overline{EI})}{\sum_{j=0}^{n}{({UV}_{j} - \overline{UV})}^{2}}.$$

Similarly, the MDR, calculated as the slope of MI versus UV dosage on D7, was also defined and utilized for the evaluation of tanning response as below:

$$\mathrm{MDR }= \frac{\sum_{j=0}^{n}({UV}_{j} - \overline{UV})({MI}_{j} - \overline{MI})}{\sum_{j=0}^{n}{({UV}_{j} - \overline{UV})}^{2}}.$$

Meanwhile, the difference of EI at the tested site before and after UV irradiation on D1, which was denoted as ΔE, was also used to evaluate erythema change. Similarly, ΔM represented the increased level of MI on D7 after a specified UV dosage.

$$\Delta {\text{E }} = {\text{EI}}_{{{\text{uv}} - {\text{irradiation}}}} {-}{\text{ EI}}_{{{\text{un}} - {\text{irradiation}}}}$$

$$\Delta {\text{M }} = {\text{ MI}}_{{{\text{uv}} - {\text{irradiation}}}} {-}{\text{ MI}}_{{{\text{un}} - {\text{irradiation}}}}$$

A repeated-measurement analysis of variance (ANOVA) test, one-way ANOVA, and a linear regression analysis were conducted to evaluate the effects of UV dosage and time on UV-induced skin responses. The correlations between MDR and ΔM and between EDR and ΔE were analyzed with Pearson's or Spearman's correlation test when appropriate. All analyses were conducted with SPSS 19.0 (version 19.0, SPSS Inc., 2008).

## Results

### Selecting Safe and Effective Dosage for Measurement of UV-induced Skin Phenotypes

High-intensity UV can cause skin damage, with reactions ranging from mild erythema to the appearance of blisters. We investigated the safe threshold of UV dosage by using a sunlight simulator to set six UV doses of 36, 40, 45, 50, 55, and 60 mJ/cm^2^ to irradiate the skin of 31 volunteers (cohort 1) and evaluating the skin reaction 24 h after irradiation. A UV dosage was considered unsafe if serious adverse skin reactions such as edema or blisters occurred.

**Fig. 1:**
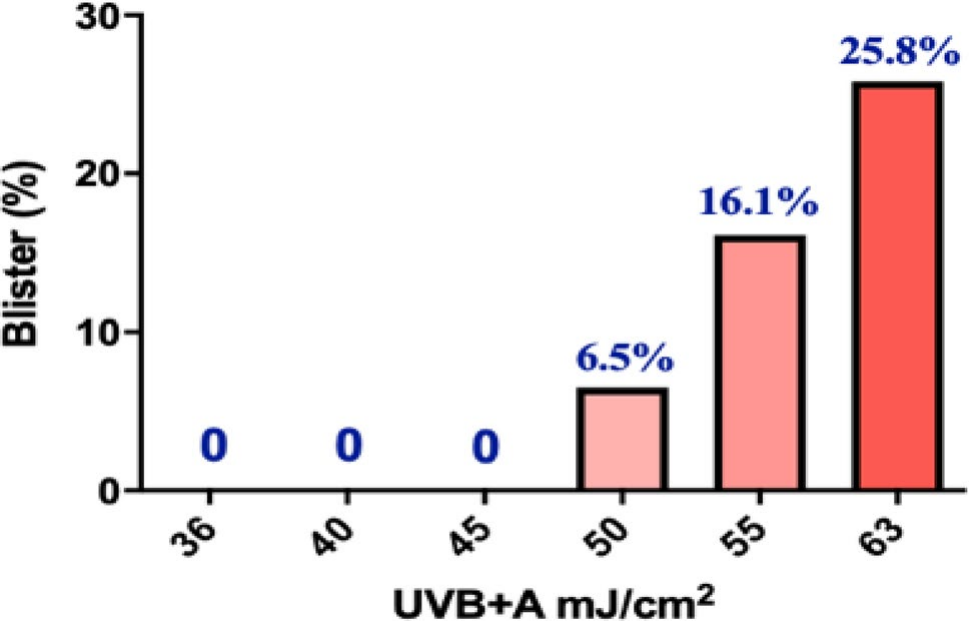
The safe dosage threshold for eliciting UV-induced skin phenotypes. The panel shows the proportions of skin blisters after different dosages of UV. The blister proportion was calculated by 31 individuals

As shown in Fig. 1, the proportions of skin blisters caused by UV irradiation were dosage-dependent, and the UV dosage of 50 mJ/cm^2^ or above could significantly increase the occurrence of skin blisters (50 mJ/cm^2^, 6.5%; 55 mJ/cm^2^, 16.1%; 60 mJ/cm^2^, 25.8%). In contrast, we did not observe any skin blisters at dosages of 45 mJ/cm^2^ or below. Therefore, dosages of no more than 45 mJ/cm^2^ are relatively safe for measuring UV-induced skin responses in the Han Chinese population. In addition, we observed significant erythema in all subjects at 45 mJ/cm^2^, suggesting that a UVB + A dose of 45 mJ/cm^2^ is both relatively safe and can induce significant skin reactions.

### Selecting Time Points for Measurement of UV- Induced Skin Phenotypes

**Fig. 2:**
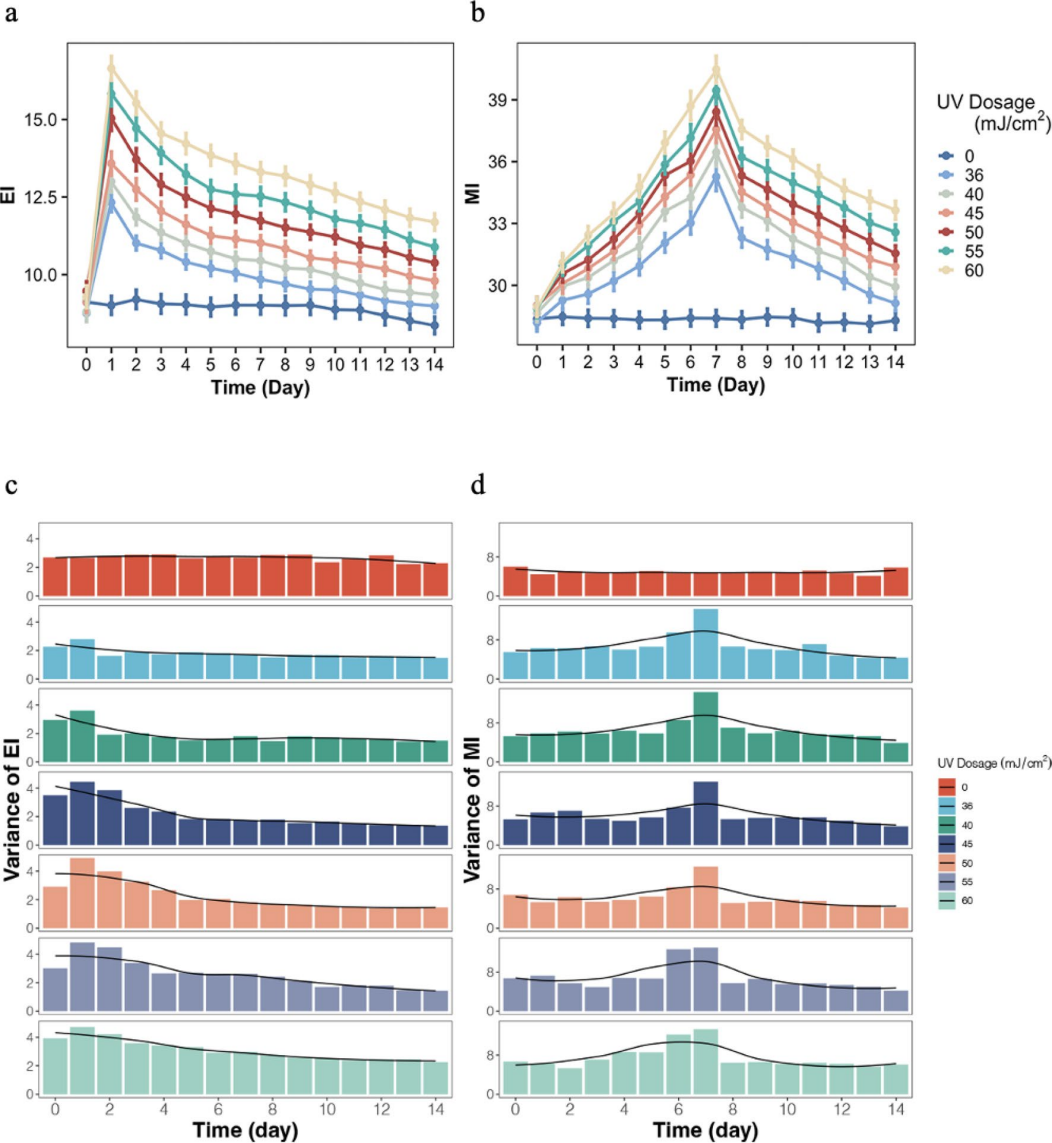
The change of EI and MI after different dosages of UV irradiation treatment. **a** and **b** present EI and MI, respectively, within 14 days after irradiation for six gradient UV dosages. The baseline values were set as the measured EI and MI of the unirradiated (0 mJ/cm^2^) skin each day. The error bars represent the standard error of each measurement. EI and MI peaked on D1 and D7, respectively, regardless of UV dosage. **c** and **d** show the variance of EI and MI, respectively, from D1 to D14 after UV irradiation. The variances of EI and MI peaked on D1 and D7, respectively

To further observe the characteristics of skin response over time after UV irradiation, we measured the EI and MI values before and after UV irradiation on the back skin of 31 volunteers in cohort 1 for 14 consecutive days. We found that both EI and MI were significantly associated with dosage and time. As shown in Fig. 2a, EI increased significantly and peaked on D1 after UV irradiation regardless of dosage (*p* < 0.001), and then gradually decreased. The MI peaked on D7 regardless of dosage (*p* < 0.001) (Fig. 2b). We also examined whether age or gender would affect the changes of EI and MI after UV irradiation. As shown in Fig. S1, neither gender nor age affected the changing pattern of EI and MI over time after UV irradiation. Further analysis showed that the variance of EI peaked on D1 and then decreased gradually (Table S2, Fig. 2c). Similarly, the variance of MI peaked at D7, which was more than twice the baseline level (Table S3, Fig. 2d). Because higher phenotype diversity could better distinguish the subtle differences between individuals, the most appropriate time points for measuring erythema and tanning responses would be the first and seventh day after UV irradiation, respectively. Therefore, we proposed that measurements of erythema and tanning responses after UV irradiation be performed on D1 and D7, respectively. These measurement time points are consistent with the time points for clinical assessment of erythema and tanning in previous study (Wagner et al. 2002a).

### Selecting Characteristic Parameters for Measurement of UV-induced Skin Phenotypes

We have found that 45 mJ/cm^2^ is a relatively safe UV dosage, but it is still unclear whether it is the appropriate dosage to quantify erythema and tanning responses. To this end, we analyzed the differences between changes in erythema and tanning responses after different UV dosages. EDR and MDR are two objective measurements commonly used to characterize the erythema and tanning responses, and EDR and MDR are based on the slopes of EI and MI versus UVR dosage. We calculated four EDRs using 45 mJ/cm^2^, 50 mJ/cm^2^, 55 mJ/cm^2^, and 60 mJ/cm^2^ as the maximum dosages and found EDR_45_ was linearly correlated with EDRs with higher maximum dosages (EDR_45_ vs. EDR_50_, *R*^2^ = 0.94; EDR_45_ vs. EDR_55_, *R*^2^ = 0.90; EDR_45_ vs. EDR_60_, *R*^2^ = 0.83), suggesting that EDR_45_ could both generate similar results and avoid the occurrence of skin blisters (Table S4). Concordantly, MDR_45_ was also linearly correlated with MDRs with higher dosages (MDR_45_ vs. MDR_50_, *R*^2^ = 0.97; MDR_45_ vs. MDR_55_, *R*^2^ = 0.90; MDR_45_ vs. MDR_60_, *R*^2^ = 0.80). Taken together, both EDR and MDR could be accurately measured with the safety UVR dosage of 45 mJ/cm^2^ as the maximum dosage (Table S4).

In addition to EDR and MDR, ΔE and ΔM are also used for quantifying the erythema and tanning responses. We calculated the ΔE and ΔM of each dosage on D1 and D7, respectively, and assessed the correlations between ΔE_45_ and ΔE values of other dosages. As shown in Table S5, significant correlations between ΔE_45_ and each ΔE of other dosages were identified (*R*^2^ > 0.75, *p* < 0.0001). Similarly, ΔM_45_ was also significantly correlated with ΔM values of other dosages (*R*^2^ > 0.75, *p* < 0.0001). These results suggested that both ΔE_45_ and ΔM_45_ are accurate measurements of UV-induced erythema and tanning phenotypes at the safe UV dosage.

**Fig. 3:**
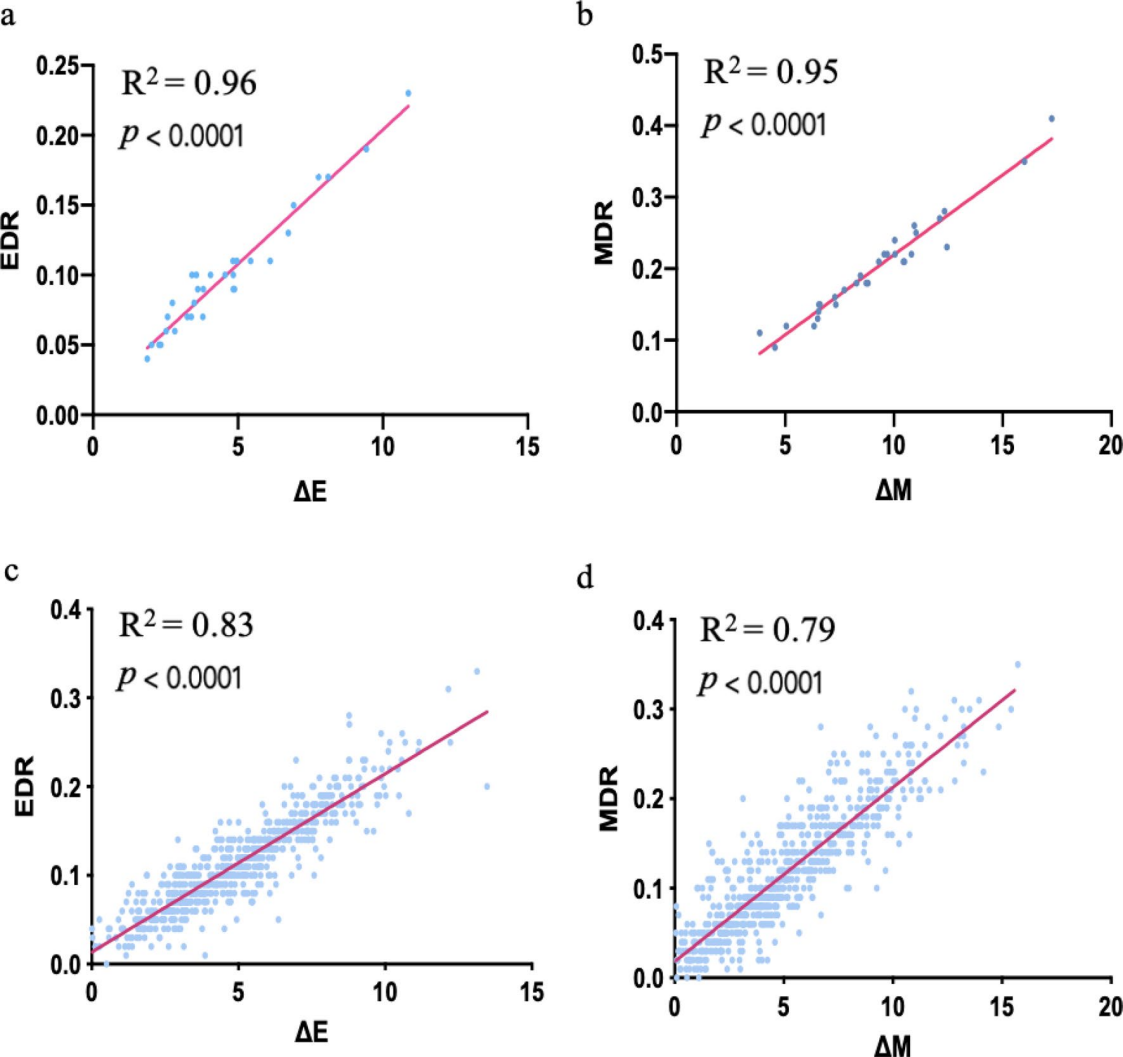
Comparison of the two group indexes of UV-induced skin phenotypes. The correlations between ΔE and EDR and between ΔM and MDR were calculated. The *R*^2^ value and *p* value were calculated through the Pearson's correlation test. Panels **a** and **b** were calculated based on 31 individuals, and panels **c** and **d** were measured based on 664 individuals

Unlike EDR and MDR, ΔE and ΔM are based on a single dosage and are easy to implement in large-sample studies. Therefore, we assessed their correlations with EDR and MDR, respectively. As shown in Fig. 3a, EDR_45_ and ΔE_45_ were linearly correlated (*R*^2^ = 0.96, *p* < 0.0001), and a similar highly linear relationship was also found between MDR_45_ and ΔM_45_ (*R*^2^ = 0.95, *p* < 0.0001) (Table S6, Fig. 3b). Based on the significant correlations between these two sets of measurements, ΔE_45_ and ΔM_45_ are appropriate and adequate alternatives to EDR_45_ and MDR_45_ for assessing UV-induced erythema and tanning phenotypes with safety, accuracy, and simplicity. To exclude the potential effects of age, gender, MED, occupation exposure, and skin phototypes on the correlations between these two sets of measurements, we performed partial correlation analysis with these factors as covariates, and we found that the two sets of measurements were significantly correlated regardless of the covariates (Table S7).

### Proposing an Assessment Procedure for Measurement of UV-induced Skin Phenotypes

Based on the results of the cohort 1 study, we proposed that the UV-induced phenotypic erythema response and tanning ability of the skin should be measured as follows.

- The irradiation light source should be a sunlight simulator in the UVB + A band with a maximum single exposure dose of 45 mJ/cm^2^.
- The UV-induced erythema response phenotype should be measured on D1 after irradiation, and the phenotypic parameter ΔE (EDR) should be derived from the EI value.
- The UV-induced tanning ability phenotype should be measured on D7 after irradiation, and the phenotypic parameter selection ΔM (MDR) should be derived from the MI values.

### Using the Independent Cohort to Validate the Assessment Procedure for UV-induced Skin Phenotypes

To validate the reliability of the phenotypic measures of UV-induced erythema and tanning ability, we recruited another independent cohort of the Han Chinese population with a large sample size (N = 664). The results in the discovery stage revealed that a dosage of 45 mJ/cm^2^ is relatively safe for measuring UV-induced erythema and tanning responses. Therefore, the UV dosages were set as 20, 25, 30, 36, 40, and 45 mJ/cm^2^ in the validation stage. One day after irradiation, we found that none of the irradiated sites developed blisters, but all developed a clearly visible erythematous reaction at the highest dose (45 mJ/cm^2^). These results further indicated that 45 mJ/cm^2^ is a safe and effective dose for evaluating skin erythema and tanning response. In addition, as expected, significant correlations between the two sets of measurements (ΔE vs. EDR and ΔM vs. MDR) were found (*R*^2^ > 0.79), indicating that ΔE and ΔM are ideal alternative measurements for erythema and tanning ability, respectively (Fig. 3c–d).

## Discussion

UV-induced skin responses, especially the erythema and tanning responses, show great diversity within and between populations, and such features of East Asians remain unclear due to lack of study (Jablonski and Chaplin 2013; Tadokoro et al. 2005; Wagner et al. 2002b). Clinical testing is an effective way to accurately quantify UV-induced skin phenotypes. However, excessive UV irradiation can produce severe skin damage, such as blistering, which may seriously affect the precise measurement of UV-induced phenotypes. Therefore, it is crucial to determine the UV dosage that will not cause serious adverse skin reactions but will induce visible skin erythema and tanning phenotypes in clinical studies. To this end, we treated 31 volunteers with different UV dosages and found that UV dosages higher than 45 mJ/cm^2^ could induce severe skin blisters. In particular, the proportion of skin blister occurrence reached 25.8% when the UV dosage reached 60 mJ/cm^2^. In addition, we further verified that irradiation at the dosage of 45 mJ/cm^2^ did not cause serious adverse skin reactions but induced significant skin erythema and tanning reactions in a larger cohort. Collectively, these results confirmed that 45 mJ/cm^2^ is an appropriate, relatively safe threshold dosage for studying UV-induced erythema and tanning phenotypes in the Chinese population. Dosages between 41 and 45 mJ/cm^2^ might also be appropriate doses for inducing erythema and tanning phenotypes, but this will require further exploration. It should also be noted that the samples used in this study are all from the Han Chinese population, and further studies are required to verify these findings in other ethnic populations.

In clinical practice, UV-induced erythema and tanning response are generally assessed on D1 and D7 after UV irradiation. However, these time points are usually chosen for convenience and lack a theoretical basis. To select the ideal measurement time points, we completed 14 consecutive days of measurements of EI and MI values after serial doses of UV irradiation. It was revealed that the erythema response peaked on D1 after UV irradiation and the tanning response peaked on D7 after UV irradiation, which is consistent with most previous studies (Kohli et al. 2019; Park et al. 2002; Suh et al. 2007). In our study, the peaks of the erythema and tanning responses occurred within 24 h and 6–8 days, respectively, but future studies should further narrow down the precise timing of their peaks.

Moreover, we confirmed that the dosage of 45 mJ/cm^2^ could induce comparable skin responses to higher UV dosages. Although both erythema and tanning responses after UV exposure are signs of skin damage, serious adverse reactions such as blistering or edema, which are induced by excessive UV radiation, can lead to skin diseases and also affect skin color measurements. Therefore, we considered a UV dosage that does not produce blistering and edema as a relatively safe dose in this study. We also found that the variances of EI and MI peaked on D1 and D7 after UV irradiation, respectively (Fig. 2c–d, Table S2–S3). These results suggest that measurements on D1 and D7 after UV irradiation may facilitate the accurate quantification of erythema and tanning responses.

The dose–response curves, including EDR and MDR, have long been considered the best evaluation measures of erythema and tanning responses. However, the laborious procedure of acquiring dose–response curves has limited their application in large population-based studies. Herein, we proposed simplified and objective measurement indexes, ΔE and ΔM, as alternatives to facilitate the evaluation of UV-induced responses. We found significant correlations between EDR and ΔE (*R*^2^ > 0.90) as well as between MDR and ΔM (*R*^2^ > 0.90) under each UV dosage in our cohorts. Our partial correlation analysis showed that other covariates, such as age, gender, occupational exposure, MED, and skin phototype, had little effect on the correlation between these two sets of measurements. Therefore, we did not include information on MED, skin phototype, or occupational exposure in cohort 2 for analysis.

Considering the accuracy, convenience, and safety of the measurements, ΔE and ΔM assessed on days 1 and 7 after UV irradiation, respectively, with the safety dosage of 45 mJ/cm^2^ could be optimal measurements for evaluating erythema and tanning responses. In this study, we developed a simplified and precise method to quantify UV-induced erythema and tanning responses, which will provide an important basis for resolving the genetic mechanisms of UV-induced skin responses in large-scale studies and developing anti-photoaging products in the future.

## Conclusion

In summary, the ΔE on D1 and ΔM on D7 after UV irradiation at the relatively safe UV dosage of 45 mJ/cm^2^ are ideal measurements for UV-induced erythema and tanning ability, respectively.

### Supplementary Information

Below is the link to the electronic supplementary material.

Supplementary file1 (DOCX 593 kb)

## Data Availability

The authors declare that all data supporting the findings of this study are available within the article.

## Abbreviations

UVR: Ultraviolet radiation
EI: Erythema index
MI: Melanin index
EDR: Erythema dose–response
MDR: Melanin dose–response
ΔE: Erythema increment
ΔM: Melanin increment
UV: Ultraviolet
MED: Minimum erythema dosage
