# Supplementary material for: Quantitative Assessment of Ultraviolet-Induced Erythema and Tanning Responses in the Han Chinese Population
Source: Phenomics. 2023 Jun 5;4(2):138–45. doi: 10.1007/s43657-023-00105-1 (PMC11169260; doi:10.1007/s43657-023-00105-1)
Supplement: Supplementary file 1 — Supplementary file1 (DOCX 593 kb) [file 43657_2023_105_MOESM1_ESM.docx]

**Supplementary Materials**

**
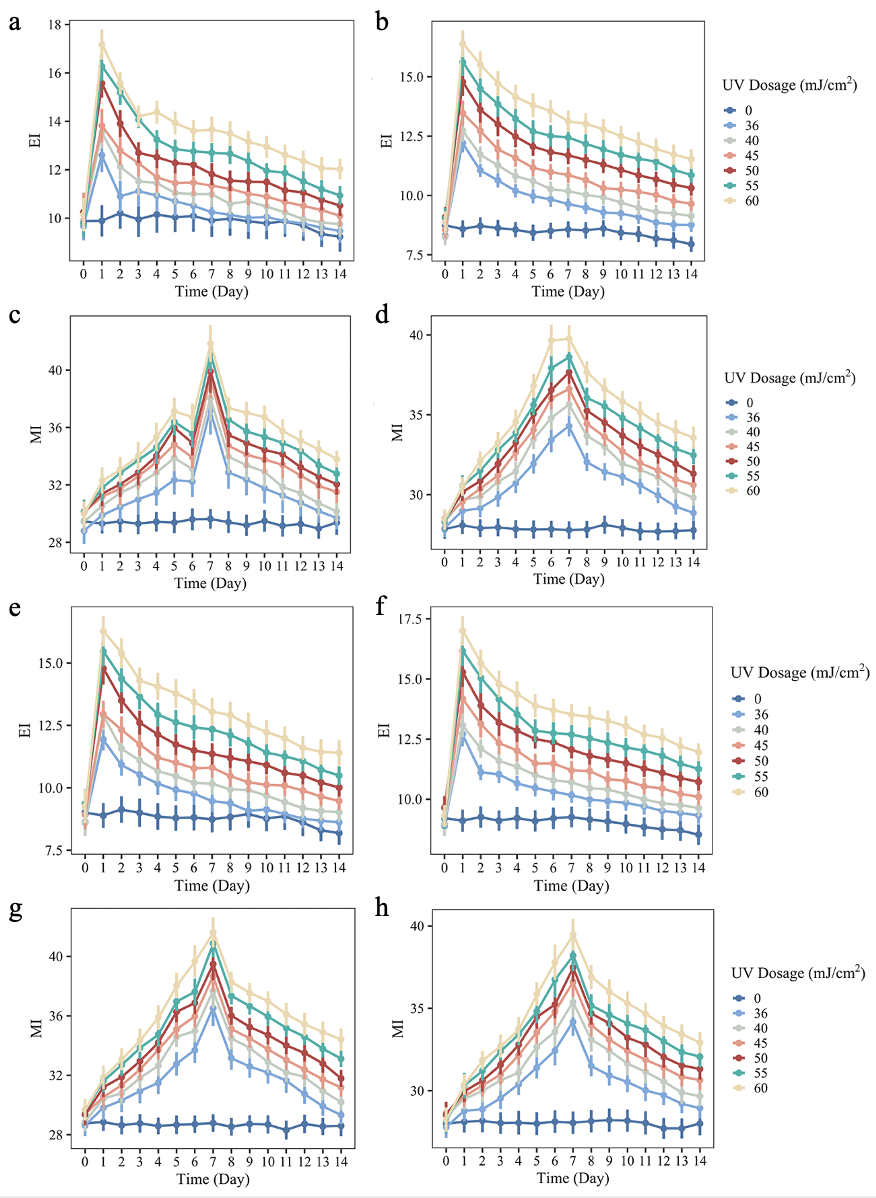
**

Fig.S1 The change of EI and MI after different dosages of UV irradiation treatment. (a) EI of male subjects. (b) EI of female subjects. (c) MI of male subjects. (d) MI of female subjects. (e) EI of the high age group. (f) EI of the low age group. (g) MI of the high age group. (h) MI of the low age group

| **Table S1.** Demographic information   \| Subject No. \| Gender \| Age (years) \| Occupational sun exposure \| MED \| Fitzpatrick photo-type scale \| \| --- \| --- \| --- \| --- \| --- \| --- \| \| 1 \| Female \| 40 \| Never \| 27.5 \| 2 \| \| 2 \| Female \| 53 \| Never \| 25 \| 3 \| \| 3 \| Female \| 41 \| Never \| 30 \| 3 \| \| 4 \| Male \| 57 \| Never \| 25 \| 3 \| \| 5 \| Male \| 47 \| Never \| 30 \| 3 \| \| 6 \| Female \| 38 \| Never \| 36 \| 2 \| \| 7 \| Female \| 47 \| Never \| 36 \| 3 \| \| 8 \| Female \| 33 \| Never \| 25 \| 3 \| \| 9 \| Male \| 48 \| Never \| 36 \| 4 \| \| 10 \| Female \| 42 \| Never \| 33 \| 3 \| \| 11 \| Male \| 48 \| Never \| 25 \| 3 \| \| 12 \| Female \| 54 \| Never \| 27.5 \| 3 \| \| 13 \| Male \| 30 \| Never \| 25 \| 3 \| \| 14 \| Female \| 32 \| Never \| 25 \| 3 \| \| 15 \| Female \| 48 \| Sometimes \| 25 \| 3 \| \| 16 \| Male \| 35 \| Never \| 36 \| 4 \| \| 17 \| Female \| 41 \| Never \| 30 \| 3 \| \| 18 \| Female \| 57 \| Never \| 27.5 \| 3 \| \| 19 \| Female \| 58 \| Never \| 25 \| 3 \| \| 20 \| Female \| 44 \| Never \| 25 \| 3 \| \| 21 \| Female \| 32 \| Never \| 25 \| 3 \| \| 22 \| Male \| 32 \| Never \| 27.5 \| 4 \| \| 23 \| Female \| 46 \| Never \| 36 \| 3 \| \| 24 \| Female \| 57 \| Never \| 25 \| 3 \| \| 25 \| Female \| 44 \| Sometimes \| 25 \| 3 \| \| 26 \| Female \| 38 \| Never \| 27.5 \| 3 \| \| 27 \| Male \| 29 \| Never \| 36 \| 4 \| \| 28 \| Female \| 57 \| Never \| 25 \| 3 \| \| 29 \| Male \| 57 \| Never \| 30 \| 4 \| \| 30 \| Female \| 54 \| Never \| 33 \| 2 \| \| 31 \| Male \| 32 \| Never \| 40 \| 3 \|   **Table S2**. Variance of the EI after gradient UVR | | | | | | | |
| --- | --- | --- | --- | --- | --- | --- | --- | --- | --- | --- | --- | --- | --- | --- | --- | --- | --- | --- | --- | --- | --- | --- | --- | --- | --- | --- | --- | --- | --- | --- | --- | --- | --- | --- | --- | --- | --- | --- | --- | --- | --- | --- | --- | --- | --- | --- | --- | --- | --- | --- | --- | --- | --- | --- | --- | --- | --- | --- | --- | --- | --- | --- | --- | --- | --- | --- | --- | --- | --- | --- | --- | --- | --- | --- | --- | --- | --- | --- | --- | --- | --- | --- | --- | --- | --- | --- | --- | --- | --- | --- | --- | --- | --- | --- | --- | --- | --- | --- | --- | --- | --- | --- | --- | --- | --- | --- | --- | --- | --- | --- | --- | --- | --- | --- | --- | --- | --- | --- | --- | --- | --- | --- | --- | --- | --- | --- | --- | --- | --- | --- | --- | --- | --- | --- | --- | --- | --- | --- | --- | --- | --- | --- | --- | --- | --- | --- | --- | --- | --- | --- | --- | --- | --- | --- | --- | --- | --- | --- | --- | --- | --- | --- | --- | --- | --- | --- | --- | --- | --- | --- | --- | --- | --- | --- | --- | --- | --- | --- | --- | --- | --- | --- | --- | --- | --- | --- | --- | --- | --- | --- | --- | --- | --- | --- | --- | --- | --- | --- | --- |
| Time (day) | UV dosage (mJ/cm^2^) | | | | | | |
|  | 0 | 36 | 40 | 45 | 50 | 55 | 60 |
| 0 | 2.70 | 2.26 | 2.95 | 3.50 | 2.91 | 3.01 | 3.92 |
| 1 | 2.68 | **2.79** | **3.61** | **4.44** | **4.91** | **4.81** | **4.71** |
| 2 | 2.78 | 1.62 | 1.92 | 3.85 | 3.98 | 4.48 | 4.21 |
| 3 | 2.88 | 1.89 | 2.02 | 2.61 | 3.25 | 3.38 | 3.58 |
| 4 | 2.91 | 1.73 | 1.76 | 2.35 | 2.67 | 2.65 | 3.42 |
| 5 | 2.64 | 1.87 | 1.51 | 1.83 | 1.97 | 2.70 | 3.31 |
| 6 | 2.74 | 1.81 | 1.60 | 1.83 | 2.05 | 2.62 | 2.92 |
| 7 | 2.69 | 1.73 | 1.80 | 1.76 | 1.82 | 2.62 | 2.97 |
| 8 | 2.86 | 1.52 | 1.47 | 1.79 | 1.68 | 2.42 | 2.76 |
| 9 | 2.88 | 1.68 | 1.79 | 1.54 | 1.65 | 2.12 | 2.56 |
| 10 | 2.36 | 1.67 | 1.72 | 1.65 | 1.50 | 1.70 | 2.52 |
| 11 | 2.60 | 1.50 | 1.64 | 1.51 | 1.50 | 1.82 | 2.38 |
| 12 | 2.84 | 1.60 | 1.60 | 1.41 | 1.39 | 1.79 | 2.45 |
| 13 | 2.24 | 1.59 | 1.43 | 1.38 | 1.45 | 1.47 | 2.45 |
| 14 | 2.30 | 1.48 | 1.50 | 1.37 | 1.45 | 1.43 | 2.26 |

| **Table S3**. Variance of the MI over a 14-day follow-up after gradient UVR | | | | | | | |
| --- | --- | --- | --- | --- | --- | --- | --- |
| Time (day) | UV dosage (mJ/cm^2^) | | | | | | |
|  | 0 | 36 | 40 | 45 | 50 | 55 | 60 |
| 0 | 5.96 | 5.48 | 5.27 | 5.27 | 6.80 | 6.70 | 6.68 |
| 1 | 4.42 | 6.32 | 5.86 | 6.64 | 5.32 | 7.27 | 6.29 |
| 2 | 4.98 | 6.30 | 6.23 | 7.01 | 6.31 | 5.72 | 5.31 |
| 3 | 4.67 | 6.67 | 5.84 | 5.37 | 5.40 | 4.94 | 7.08 |
| 4 | 4.76 | 6.01 | 6.41 | 4.96 | 5.76 | 6.80 | 8.63 |
| 5 | 5.09 | 6.59 | 5.85 | 5.67 | 6.45 | 6.65 | 8.55 |
| 6 | 4.65 | 9.52 | 8.57 | 7.66 | 8.36 | 12.65 | 12.19 |
| 7 | 4.77 | **14.31** | **14.30** | **12.95** | **12.58** | **13.00** | **13.30** |
| 8 | 4.67 | 6.62 | 7.00 | 5.31 | 5.14 | 5.74 | 6.44 |
| 9 | 4.80 | 6.11 | 5.89 | 5.58 | 5.40 | 6.60 | 6.59 |
| 10 | 4.77 | 5.91 | 6.34 | 5.68 | 5.80 | 5.54 | 6.21 |
| 11 | 5.16 | 7.12 | 5.68 | 5.68 | 5.57 | 5.68 | 6.44 |
| 12 | 4.66 | 4.80 | 5.59 | 4.98 | 4.69 | 5.35 | 6.23 |
| 13 | 4.12 | 4.31 | 5.27 | 4.43 | 4.76 | 5.01 | 5.61 |
| 14 | 5.79 | 4.35 | 3.89 | 3.83 | 4.19 | 4.22 | 6.05 |

| **Table S4.** Comparisons of correlation between different EDRs or MDRs at different maximum doses | | |
| --- | --- | --- |
| Comparison | *R*^2^ | Significance |
| EDR_45_ vs. EDR_50_ | 0.94 | *p* < 0.0001 |
| EDR_45_ vs. EDR_55_ | 0.90 | *p* < 0.0001 |
| EDR_45_ vs. EDR_60_ | 0.83 | *p* < 0.0001 |
| MDR_45_ vs. MDR_50_ | 0.97 | *p* < 0.0001 |
| MDR_45_ vs. MDR_55_ | 0.90 | *p* < 0.0001 |
| MDR_45_ vs. MDR_60_ | 0.80 | *p*< 0.0001 |

EDR_45_ was calculated with four doses: 0, 36, 40, and 45 mJ/cm^2^; EDR_50_ was calculated with five doses: 0, 36, 40, 45, and 50 mJ/cm^2^; EDR_55_ was calculated with six doses: 0, 36, 40, 45, 50, and 55 mJ/cm^2^; EDR_60_ was calculated with seven doses: 0, 36, 40, 45, 50, 55, and 60 mJ/cm^2^. The calculation method of MDR was the same as EDR. The *R*^2^ and significance were calculated through the Pearson’s correlation test.

| **Table S5.** Comparisons of correlation between color changes after different UV dosages | | |
| --- | --- | --- |
| Comparison | *R*^2^ | Significance |
| ΔE_45_ vs. ΔE_40_ | 0.81 | *p* < 0.0001 |
| ΔE_45_ vs. ΔE_50_ | 0.81 | *p* < 0.0001 |
| ΔE_45_ vs. ΔE_55_ | 0.78 | *p* < 0.0001 |
| ΔE_45_ vs. ΔE_60_ | 0.78 | *p* < 0.0001 |
| ΔM_45_ vs. ΔM_40_ | 0.81 | *p* < 0.0001 |
| ΔM_45_ vs. ΔM_50_ | 0.92 | *p* < 0.0001 |
| ΔM_45_ vs. ΔM_55_ | 0.84 | *p* < 0.0001 |
| ΔM_45_ vs. ΔM_60_ | 0.76 | *p* < 0.0001 |

| **Table S6.** Comparisons of correlation between dosage curves and color changes | | |
| --- | --- | --- |
| Comparison | *R*^2^ | Significance |
| EDR_40_ vs. ΔE_40_ | 0.92 | *p* < 0.0001 |
| EDR_45_ vs. ΔE_45_ | 0.96 | *p* < 0.0001 |
| EDR_50_ vs. ΔE_50_ | 0.96 | *p* < 0.0001 |
| EDR_55_ vs. ΔE_55_ | 0.95 | *p* < 0.0001 |
| EDR_60_ vs. ΔE_60_ | 0.94 | *p* < 0.0001 |
| MDR_40_ vs. ΔM_40_ | 0.97 | *p* < 0.0001 |
| MDR_45_ vs. ΔM_45_ | 0.95 | *p* < 0.0001 |
| MDR_50_ vs. ΔM_50_ | 0.98 | *p* < 0.0001 |
| MDR_55_ vs. ΔM_55_ | 0.95 | *p* < 0.0001 |
| MDR_60_ vs. ΔM_60_ | 0.96 | *p* < 0.0001 |

**Table S7.** Comparisons of correlation between dosage curves and color changes with gender, age, occupational sun exposure, MED, and Fitzpatrick photo-type scale as covariates

| Parameters | Partial correlation coefficient | *p*-value |
| --- | --- | --- |
| MDR vs. ΔM | 0.97 | 0.43×10^-15^ |
| EDR vs. ΔE | 0.98 | 0.89×10^-17^ |
